# Disparities in Sex-Based Health Outcomes of Endovascular Intervention for the Treatment of Symptomatic Lower Extremity Peripheral Artery Disease: A Scoping Review

**DOI:** 10.7759/cureus.111351

**Published:** 2026-06-23

**Authors:** Lila Dudley, Mackenzie Morse, Julia L Armstrong, Brooke L Heyer, Jonathan Platosz, Shannon N Smith, Sierra Scott, Sydney L Elness, Myroslava Ljavenec, Aidan Reed, Suzanne I Riskin

**Affiliations:** 1 Department of Foundational Sciences, Nova Southeastern University Dr. Kiran C. Patel College of Osteopathic Medicine, Clearwater, USA

**Keywords:** angioplasty, atherectomy, endovascular angioplasty, lower extremity peripheral arterial disease, lower limb, peripheral angioplasty, peripheral arterial disease, peripheral arterial disease and peripheral angioplasty, stent, vascular stent

## Abstract

Peripheral artery disease (PAD) is a chronic vascular condition that affects millions of individuals worldwide and is a significant burden on public health. Although female subjects may be at greater risk of developing PAD owing to sex-specific risk factors and frequently present with atypical symptoms, diagnosis rates are comparable between women and men. Treatment of PAD commonly relies on endovascular interventions, including angioplasty, stenting, and atherectomy. While these interventions have proven effective, understanding sex-specific differences in outcomes has been insufficiently studied. This scoping review was conducted to assess the health outcomes of endovascular interventions for PAD in female subjects residing in the United States (US). A systematic search was performed across multiple databases, including EMBASE, Ovid MEDLINE, and Web of Science. The inclusion criteria focused on studies published between 2016 and 2024, involving lower extremity PAD interventions across the US female populations. Studies evaluating non-US populations, pharmacological treatments, and asymptomatic individuals were excluded. Data extraction focused on key factors, including (1) complication rates, (2) need for reintervention, and (3) long-term health outcomes.

Nine retrospective cohort studies met the inclusion criteria. We analyzed complications, reintervention rates, amputation risks, and mortality outcomes, revealing variation in findings. Five studies reported increased complications in female patients, such as arterial dissection and hematoma formation. Additionally, four studies showed higher rates of reintervention among female patients, while one found no significant sex-based differences in reintervention or complications. Similarly, while three studies identified a greater risk of amputation and mortality in female patients, two others revealed no disparities or better outcomes among female patients. The variability in outcome measures, study designs, and patient populations may have contributed to the inconsistencies across these findings. These varied findings highlight the need for more standardized research measures to accurately assess how sex-based differences impact PAD treatment outcomes. Further research is necessary to explore anatomical, physiological, and sociocultural factors contributing to these disparities and to improve treatment strategies for female patients with PAD.

## Introduction and background

Peripheral artery disease (PAD) poses a significant risk to public health and has been shown to impact over 230 million individuals worldwide and 8.5 million within the United States (US) [1,2]. PAD results from progressive arterial narrowing due to plaque accumulation, resulting in reduced blood flow and oxygen delivery [3]. Risk factors for disease progression include increasing age, hypertension, dyslipidemia, diabetes, and social risk factors, including smoking, obesity, and alcohol use [4-6]. Patients with etiological and/or social risk factors tend to experience dysregulation of inflammatory processes, increased platelet activation, and maladaptive responses, which can impair vessel tone and ultimately lead to the development of PAD and an increased risk of limb ischemia, amputation, thrombosis, and death [2,7].

PAD is diagnosed by performing an ankle-brachial index (ABI) test, comparing the blood pressure in the ankles to the blood pressure in the arms, with values less than 0.90 at rest indicating disease. Additional diagnostic tests include blood tests, Doppler ultrasound, and segmental Doppler pressure testing [8]. Management for lower extremity PAD includes both open surgical and endovascular methods. Primary surgical treatment strategies for lower extremity PAD include revascularization, angioplasty, stent placement, atherectomy, and artery bypass grafting. An endovascular approach tends to be less invasive, resulting in decreased length of stay and fewer complications, contributing to a recent shift away from open surgical interventions [9,10]. Goals for endovascular approaches include relieving intermittent claudication along with acute and critical limb ischemia resulting from PAD [11].

While sex-based differences between male and female subjects with PAD have been recognized in the literature, major knowledge gaps exist in terms of sex-specific health status and treatment outcomes [12]. Female patients have an approximately 30% higher risk of developing PAD compared with men, despite similar overall diagnosis rates between the sexes [13-16]. In addition, female patients with PAD frequently experience delayed diagnosis, often presenting 10-20 years later than male patients and with more advanced disease at the time of presentation [17]. Several factors may contribute to this delay, including atypical or asymptomatic clinical presentation, confounding musculoskeletal comorbidities such as spinal stenosis that can mimic PAD symptoms, and underlying pathophysiologic differences, including smaller arterial caliber and variations in the distribution of atherosclerotic disease [18-19].

Although PAD in female patients may be associated with a lower burden of traditional cardiovascular risk factors, its true prevalence may be underestimated. Reliance on conventional cardiovascular risk stratification tools may fail to fully capture disease severity in them, particularly given the influence of female-specific risk factors such as menopause-related hormonal changes and a history of preeclampsia [20]. In addition, current screening measures for PAD may lack adequate sex-specific sensitivity in assessing disease severity. Prior studies have demonstrated that female patients with PAD experience poorer functional status and greater ambulatory limitations than male patients at comparable ABI levels [17]. Collectively, these clinical differences may contribute to delayed disease recognition, as symptoms in female patients are more likely to be attributed to nonvascular conditions, potentially postponing appropriate vascular evaluation and diagnosis. 

Female patients with PAD have been shown to experience poorer quality of life, higher rates of depression, and an increased risk of major amputation compared with male patients [15,21,22]. Despite these reported differences, female subjects often remain underrepresented in PAD clinical trials with an average of only 33% of study participants in PAD intervention-related clinical trials from 2011-2021, despite similar diagnostic prevalence compared to males [23]. Although female patients have a lower incidence of cardiovascular disease, females with cardiovascular disease receive less care and fewer investigations [24,25]. This underrepresentation limits the ability to synthesize the discrepancies between sex- specific outcomes.

A preliminary search of the International Prospective Register of Systematic Reviews (PROSPERO), the Cochrane Database of Systematic Reviews, and PubMed conducted on September 2, 2024, revealed no current or prospective systematic or scoping reviews on this topic. Furthermore, an additional search of these databases was conducted on May 27, 2026, and revealed no scoping or systematic reviews on this topic published during this review’s development. While previous reviews have focused on sex disparities among patients with PAD, highlighting social, biological, and clinical limitations, they lacked the specificity of evaluating American female subjects and their outcomes following different endovascular approaches [7,26]. One study evaluated the influence of disparities on the efficacy of endovascular procedures among patients with PAD, but only participants with Medicare claims were included, limiting its generalizability to participants with various forms of healthcare coverage [22].

Therefore, a comprehensive understanding of sex-specific outcomes following endovascular interventions for PAD is needed. Addressing this gap may allow for reduced morbidity and mortality associated with PAD. The aim of this review is to evaluate sex-based differences in post-interventional outcomes between female and male patients, including complications such as thromboembolic and hemorrhagic events, arterial dissection, amputation, and rates of reintervention. In examining these differences, the review will also consider relevant pre-interventional factors, including the endovascular technique utilized, the arterial territory treated, procedural indication, and potential disparities in baseline preoperative risk factors.

## Review

Methods

The research question was created in accordance with the Population, Concept, and Context (PCC) strategy, establishing American female subjects in the US as the population, endovascular treatment as the concept, and PAD as the context. Based on these variables, the primary question was ‘What are the health outcomes of endovascular lower extremity intervention for the treatment of PAD among female patients within the US?’

A comprehensive search strategy was developed to identify relevant articles by analyzing key terms from titles, abstracts, and indexing terms used to describe the articles across databases such as EMBASE, Ovid MEDLINE, and Web of Science. The strategy, including all relevant keywords and indexing terms, was adapted to each specific database and information source. Controlled terms included “peripheral artery disease; angioplasty, balloon; atherectomy; cardiovascular stent; rotational/ orbital/directional atherectomy; lower limb and lower extremity.” The terms were combined using Boolean operators “AND” and “OR,” aiming to broaden the search.

For the search strategy, only studies published in English between 2016 and 2024 were considered for the subsequent screening process. This scoping review included both experimental and quasi-experimental study designs, such as randomized controlled trials, non-randomized controlled trials, before-and-after studies, and interrupted time-series studies. In addition, analytical observational studies, including prospective and retrospective cohort studies, case-control studies, and analytical cross-sectional studies, were also considered to ensure no relevant publications were overlooked.

The search identified a total of 632 articles: 325 from EMBASE, 167 from Ovid MEDLINE, and 140 from Web of Science (Tables 1-3). 

**Table 1:** EMBASE search strategy Completed on 09/13/2024, giving 325 results.

1	'peripheral arterial disease'/exp	76,767
2	('peripheral arter* diseas*':ab,ti,kw OR 'arter* occlusive diseas*':ab,ti,kw OR 'arter* obliterative diseas*':ab,ti,kw OR 'obliterative arter* diseas*':ab,ti,kw OR 'obstructive arter* disease':ab,ti,kw OR 'occlusive arter* diseas*':ab,ti,kw OR 'chronic arter* occlusi* diseas*':ab,ti,kw OR 'chronic arter* occlusion':ab,ti,kw OR 'chronic occlusion, arter*':ab,ti,kw OR 'pad (peripheral arterial disease)':ab,ti,kw OR 'paod':ab,ti,kw OR 'peripheral arter* obstructive diseas*':ab,ti,kw OR 'peripheral arter* occlusi* diseas*':ab,ti,kw OR 'peripheral atherosclerosis':ab,ti,kw OR 'peripheral obliterative arter* diseas*':ab,ti,kw OR 'peripheral obliterative vascular diseas*':ab,ti,kw OR 'peripheral occlusive arter* diseas*':ab,ti,kw OR 'peripheral occlusive disease':ab,ti,kw OR 'peripheral vascular occlusi* diseas*':ab,ti,kw OR 'arteriosclerosis obliterans':ab,ti,kw OR 'critical limb ischemia':ab,ti,kw OR 'claudication':ab,ti,kw)	62,010
3	#1 OR #2	94,308
4	'angioplasty, balloon'/exp	37,014
5	('angioplasty, balloon':ab,ti,kw OR 'angioplasty, percutaneous transluminal':ab,ti,kw OR 'angioplasty, transluminal':ab,ti,kw OR 'balloon angioplasty':ab,ti,kw OR 'dotter artery dilatation':ab,ti,kw OR 'percutaneous angioplasty':ab,ti,kw OR 'percutaneous transluminal artery dilatation':ab,ti,kw OR 'transluminal angioplasty':ab,ti,kw OR 'transluminal artery dilatation':ab,ti,kw OR 'percutaneous transluminal angioplasty':ab,ti,kw)	24,794
6	#4 OR #5	43,986
7	‘cardiovascular stent’/exp	74,516
8	('cheatham platinum':ab,ti,kw OR 'cheatham platinum stent system':ab,ti,kw OR 'cp stent system':ab,ti,kw OR 'stent, cardiovascular':ab,ti,kw OR 'cardiovascular stent':ab,ti,kw)	381
9	#7 OR #8	74,677
10	'rotational atherectom*':ab,ti,kw OR 'orbital atherectom*':ab,ti,kw OR 'directional atherectom*':ab,ti,kw OR 'atherectom*':ab,ti,kw	6,280
11	'lower limb'/exp OR 'lower extremity':ab,ti,kw	5,97,557
12	('women':ab,ti,kw OR 'female*':ab,ti,kw OR 'woman*':ab,ti,kw OR 'girl*':ab,ti,kw)	40,30,851
13	#3 AND #11 AND #12	2,390
14	#6 AND #13	180
15	#9 AND #13	82
16	#10 AND #13	63

**Table 2:** Ovid MEDLINE search strategy Completed on 09/13/2024, giving 167 results.

1	exp Peripheral Arterial Disease/	20,001
2	('peripheral arter* diseas*'OR 'arter* occlusive diseas*' or 'arter* obliterative diseas*' or 'obliterative arter* diseas*' or 'obstructive arter* disease' or 'occlusive arter* diseas*' or 'chronic arter* occlusi* diseas*' or 'chronic arter* occlusion' or 'chronic occlusion, arter*' or 'pad' or 'paod' or 'peripheral arter* obstructive diseas*' or 'peripheral arter* occlusi* diseas*' or 'peripheral atherosclerosis' or 'peripheral obliterative arter* diseas*' or 'peripheral obliterative vascular diseas*' or 'peripheral occlusive arter* diseas*' or 'peripheral occlusive disease' or 'peripheral vascular occlusi* diseas*' or 'arteriosclerosis obliterans' or 'critical limb ischemia' or 'claudication').ab,ti,kf.	51,911
3	1 or 2	60,244
4	exp Angioplasty, Balloon/	55,362
5	('angioplasty, balloon' or 'angioplasty, percutaneous transluminal' or 'angioplasty, transluminal' or 'balloon angioplasty' or 'dotter artery dilatation' or 'percutaneous angioplasty' or 'percutaneous transluminal artery dilatation' or 'transluminal angioplasty' or 'transluminal artery dilatation' or 'percutaneous transluminal angioplasty').ab,ti,kf.	16,892
6	4 or 5	62,000
7	exp Stents/	93,003
8	('cheatham platinum' or 'cheatham platinum stent system' or 'cp stent system' or 'stent, cardiovascular' or 'cardiovascular stent').ab,ti,kf.	254
9	7 or 8	93,096
10	('rotational atherectom*' or 'orbital atherectom*' or 'directional atherectom*' or 'atherectom*').ab,ti,kf.	4,066
11	exp Lower Extremity/	192,460
12	('female*' or 'women' or 'woman' or 'girl*).ab,ti,kf.	2,732,573
13	3 and 11 and 12	987
14	6 and 13	101
15	9 and 13	52
16	10 and 13	14

**Table 3:** Web of Science search strategy Completed on 09/12/2024, giving 140 results.

1	TS=(“peripheral arter* diseas*” OR “arter* occlusive diseas*” OR “arter* obliterative diseas*” OR “obliterative arter* diseas*” OR “obstructive arter* disease” OR “occlusive arter* diseas*” OR “chronic arter* occlusi* diseas*” OR “chronic arter* occlusion” OR “chronic occlusion, arter*” OR “pad (peripheral arterial disease)” OR “paod” OR “peripheral arter* obstructive diseas*” OR “peripheral arter* occlusi* diseas*” OR “peripheral atherosclerosis” OR “peripheral obliterative arter* diseas*”w OR “peripheral obliterative vascular diseas*” OR “peripheral occlusive arter* diseas*” OR “peripheral occlusive disease” OR “peripheral vascular occlusi* diseas*” OR “arteriosclerosis obliterans” OR “critical limb ischemia” OR “claudication”)	44997
2	TS=("angioplasty, balloon" OR "angioplasty, percutaneous transluminal" OR "angioplasty, transluminal" OR "balloon angioplasty" OR "dotter artery dilatation" OR "percutaneous angioplasty" OR "percutaneous transluminal artery dilatation" OR "transluminal angioplasty" OR "transluminal artery dilatation" OR "percutaneous transluminal angioplasty" )	24291
3	TS=("cheatham platinum " OR "cheatham platinum stent system " OR "cp stent system " OR "stent, cardiovascular " OR "cardiovascular stent ")	497
4	TS=("rotational atherectom*" OR"orbital atherectom*" OR"directional atherectom*" OR"atherectom*")	6045
5	TS=(“lower limb*” OR “lower extremit*”)	134265
6	TS=(“women” OR “female*” OR “woman*” OR “girl*”)	3333072
7	#1 AND #6 AND #5	1349
8	#7 AND #2	114
9	#7 AND #3	1
10	#7 and #4	25

All identified citations were compiled and uploaded into Excel (Microsoft Corp., Redmond, WA, USA), and 206 duplicates were removed, leaving a total of 426 studies to be screened. Rayyan (Rayyan Systems Inc., Cambridge, MA, US) was then used to manage records throughout a two-stage screening process, in which the articles were evaluated using the predefined inclusion and exclusion criteria. The exclusive programs utilized for this review were Excel for article organization and data extraction, and Rayyan for article screening.

For the first tier of the screening process, all independent reviewers screened the titles and abstracts of all articles to evaluate them against the inclusion criteria. The inclusion criteria consisted of studies that took place in the U.S. between January 1, 2016, and August 1, 2024. We restricted our analysis to studies in the U.S. to standardize the population to a single country. Similarly, we limited our sample to studies published from 2016 onward to keep the data more pertinent to the current medical technology. Additional inclusion criteria included studies involving the lower extremity (popliteal and femoral arteries) and management by endovascular approaches (balloon angioplasty, stenting, and atherectomy). The exclusion criteria included case studies, case series, poster presentations, articles not published in the U.S., and articles published prior to 2016. Discrepancies between reviewers regarding article inclusion were discussed as a group until all agreed on the included articles. This process revealed a total of 36 studies for further consideration.

In the second-tier screening process, the remaining studies’ full texts were evaluated in full, and their citation details were documented by two independent reviewers. Reasons for excluding sources that did not meet the criteria were recorded and reported in the scoping review. Any disagreements during the selection process were resolved through discussion or consultation with an additional reviewer when needed. The second-tier screening process resulted in nine studies retained for analysis.

**Figure 1:**
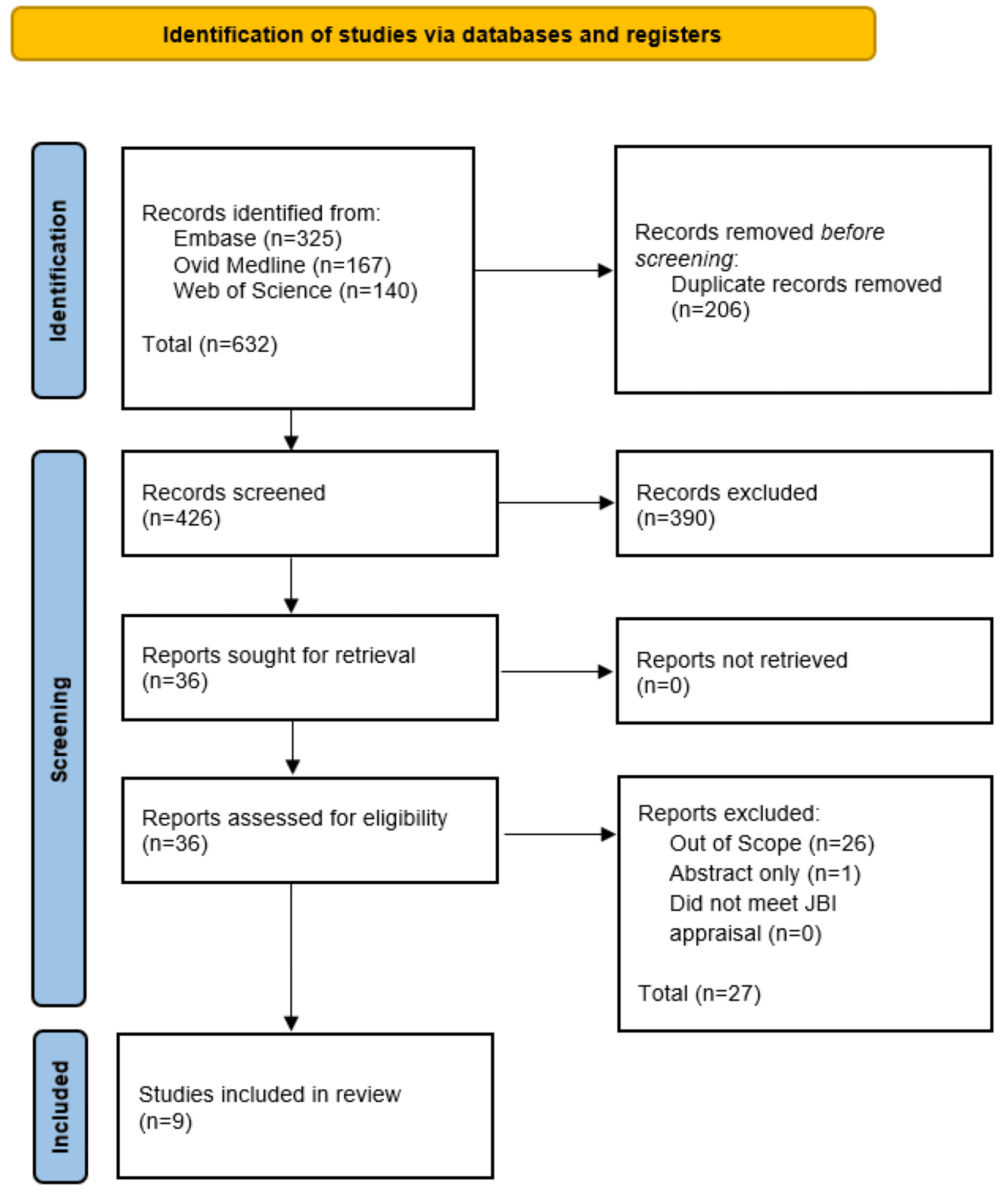
PRISMA 2020 flow diagram for new systematic reviews which included searches of databases PRISMA: Preferred Reporting Items for Systematic reviews and Meta-Analyses; JBI: Joanna Briggs Institute critical appraisal tools.

The complete search results and study selection process were reported in the final scoping review and presented in a Preferred Reporting Items for Systematic Reviews and Meta-analyses extension for scoping review (PRISMA-ScR) flow diagram shown in Figure 1 [27].

Following tier-two screening, the nine articles that met all inclusion criteria were subsequently assessed for bias and article quality with the Joanna Briggs Institute (JBI) Critical Appraisal Tools [28]. Utilizing JBI checklists for thorough assessment, each article was classified as having a high risk of bias (<50%), moderate risk of bias (50-70%), or low risk of bias (>70%) based on the criteria outlined by JBI. After detailed discussion, the nine articles were all found to have a low risk of bias and were therefore included in this scoping review. 

Following the screening process, three reviewers developed a data-charting form in Excel to determine which variables to extract from the included nine articles based on the PCC strategy. Predefined variables included study design, participants and study details, objective data, conclusions, and limitations. Reviewers independently extracted data using the standardized charting method to ensure consistency across each study. Any discrepancies in data extraction or categorization were resolved through discussion and consensus among the reviewers prior to our final analysis.

Results

Data Synthesis 

Each of the nine included papers was a retrospective cohort study conducted in the US; however, they varied in population size, objectives, conclusions, and limitations (Table 4).

**Table 4:** Summary table of the major findings from each of the nine included studies AD: arterial dissection; CTLI: chronic limb-threatening ischemia; DA: directional atherectomy; DCB: drug-coated balloon; LER: lower-extremity revascularization; NIS: National Inpatient Sampling; PAD: peripheral artery disease; PCI: percutaneous coronary intervention; PVI: peripheral vascular intervention; VQI: Vascular Quality Initiative.

Article	Study design	Participants and study details	Objective	Conclusion	Limitations
Altin et al., 2022 [29]	Retrospective cohort study	Patients acquired from VQI registry from September 2016 to March 2020; 119,620 patients; 47,316 (39.6%) females	Evaluate whether female sex is independently associated with periprocedural complication during peripheral vascular interventions	Female subjects were at a higher risk of developing access site complications and in-hospital mortality.	Limited medical history, including medications
Damara et al., 2024 [30]	Retrospective cohort study	Patients acquired from the VQI registry from 2016-2021; 117,790 cases	Evaluate the prevalence and impact of AD in patients treated for PAD with vascular interventions	AD was more common in female participants, particularly during treatment of the femoropopliteal segment, leading to increased cardiac and pulmonary complications, re-intervention, longer follow up times, and decreased patency.	Lacked information regarding the degree of severity of dissection
Levin et al., 2022 [31]	Retrospective cohort study	Patients acquired from the VQI registry from 2010-2020; 64,752 peripheral vascular intervention cases (61.9% male, 38.1% female)	Assess sex-based differences in peri-procedural and post-operative outcomes for intermittent claudication interventions	Peri-procedural complications were higher in female patients. Female patients were also more likely to require reinterventions 1 year after their surgical intervention. Researchers found no sex-based differences in mortality or amputation rates.	Limited observation period of outcomes
Ramkumar et al., 2019 [32]	Retrospective cohort study	Patients acquired from VQI registry from January 2010-October 2016; 58,247 eligible patients, 41% female	Analyze sex-based differences in arterial reintervention and occlusion rates for endovascular treatments of PAD	Females were more likely to require reintervention and develop occlusion in the femoropopliteal arteries.	Limited observation period of outcomes
Doshi et al., 2017 [33]	Retrospective cohort study	Patients acquired from the NIS database from 2012-2014; 62,444 patients included, 43.05% female	Compare gender related differences with respect to in-hospital outcomes in patients treated for PAD via endovascular approaches	Female patients experienced lower overall complication rates than males, however, presented with more urgent complications over	Increased risk of coding errors and consistency within the NIS database
Doshi et al., 2019 [34]	Retrospective cohort study	Patients acquired from NIS database from 2005-2014; 2,461,328 female patients studied, 356,092 (14.4%) received PVI	Investigate the difference between risk factors and outcomes of females who underwent PCI and PVI	A higher risk profile was associated with PVI as opposed to PCI, resulting in longer hospital stays, increased costs and worse outcomes.	Risk of outdated medical intervention and methods from the 2005-2014 cases as compared to current methods
Jeon-Slaughter et al., 2017 [35]	Retrospective cohort study	Patients acquired from XLPAD registry from January 2005 - October 2015; 1,084 patients, 449 females, prior to propensity score matching	Assess sex-based differences in 12-month outcomes following infra-inguinal endovascular procedures in patients with symptomatic PAD	Females had a statistically significant lower risk of mortality when compared to their male counterparts but required revascularization more frequently.	Small sample size. Limited observation period of outcomes
Israni et al., 2023 [36]	Retrospective cohort study	Patients acquired from a tertiary care center in New York City from 2014-2019; 294 patients, 125 female patients (42.5%)	Compare both short and long-term outcomes between males and females who had a lower extremity atherectomy for PAD	No statistically significant sex-based differences in reintervention-free survival or amputation rates	Small sample size
Hogan et al., 2023 [37]	Retrospective cohort study	Patients acquired from EPIC records from a safety net hospital from April 2016 – January 2020; 58 patients, 41% female	Examine the demographic and clinical characteristics, as well as the short-term immediate outcomes, of patients with PAD treated with DA and DCB during LER	Most patients presented with CTLI, and the DA–DCB approach yielded lower complication rates with good short-term outcomes.	Small sample size and single hospital population

Four of the nine studies utilized the Vascular Quality Initiative (VQI) database, two utilized the National Inpatient Sample (NIS) database, one utilized the Excellence in PAD (XPLAD) registry, one utilized data from a large tertiary center in New York City, and one study used data from a safety-net hospital [29-37]. Three of the nine studies solely measured periprocedural and post-operative complications following endovascular intervention, while six studies evaluated both short- and long-term complications following surgery ranging from one to four years post-intervention [25,33,34,37]. Eight of the nine studies evaluated angioplasty, atherectomy, and stent placement, with only one study evaluating atherectomy and stent placement [29-37]. The studies differed in complications evaluated and the timeline following the intervention. Given the nature of the papers included in this study as well as the study design, the following results represent narrative comparisons rather than statistical aggregation, and should be considered as such.

There was some overlap in how complications and complication rates were studied across the nine studies. Regarding complications, one of the nine studies evaluated overall complications [29]. Six of the nine studies evaluated amputation and amputation-free survival [29-31,33,36,37]. Three studies evaluated return to surgery and reintervention, including the artery treated, the procedure associated with reintervention, and the time to reintervention following initial procedure [30-32,36]. Five studies evaluated vascular complications such as hematoma formation, access site thrombosis, distal embolization, significant blood loss, and arterial dissection [29-33]. Four studies evaluated morbidity and mortality [29,31,35,37]. Five studies considered different risk factors for patients undergoing endovascular intervention to compare complication rates [29,31,35-37].

Considerations Prior to Intervention

With regards to indications for endovascular intervention, female subjects were more commonly treated for complete occlusion (32.8% of interventions) of a vessel in comparison to male subjects (27.8% of interventions) [36]. Among patients presenting with occlusion of a vessel, female patientss tended to present with chronic limb-threatening ischemia (CTLI), while male patients commonly presented with claudication [29]. Male patients were more likely to undergo endovascular intervention for stenosis of a vessel (59.9% of interventions) as compared to their female counterparts (56% of interventions) [36]. 

The most common procedure used for management of PAD among female patients was stent placement, and atherectomy procedures [29,32,35,36]. Regarding the arteries treated, studies did not identify a single vessel as consistently requiring endovascular intervention. Two studies suggested that the superficial femoral and femoropopliteal artery were the most common sites for intervention, while three indicated the iliac region was most common [29,31,32,35,36].

Prior to endovascular intervention for PAD, studies agreed that female patients tended to be older, of African American descent, and never smokers [29,31,35,36]. These female patients were also more likely to have no history of prior coronary artery disease or revascularization of the coronary arteries [31]. The risk factors that tended to be more predominant in the female cohort included obesity, need for ambulatory assistance, presence of COPD, and anemia [29,31]. Male patients tended to have higher rates of diabetes, coronary artery disease, chronic heart failure, and a history of smoking [29]. 

Risk of Complications of Reintervention

Female patients were more likely to develop overall complications following endovascular intervention at 4.2%, which was higher than their male counterparts (2.9%) [29]. This included an increased risk of access site hematomas (average rate of 3.15%) as compared to male patients (average rate of 2.05%) [29,31]. Female subjects were also more likely to have distal embolization (1.17 OR) and significant blood loss (14.9%) in comparison to male subjects (11.9%) [29,33]. In addition, female patients were more likely to have access site thrombosis as compared with male patients at the iliac and femoropopliteal arteries, with a hazard ratio of 1.42 and 1.19, respectively [32]. Meanwhile, male patients had a higher likelihood of having access site thrombosis at the tibial arteries (HR 0.89) [32]. These findings should be interpreted with caution, as they are derived from heterogeneous retrospective cohort studies.

Two studies investigated the rates of arterial dissection as a complication of peripheral vascular intervention [29,30]. One study reported that the overall risk of arterial dissection was 3% [30]. Additionally, another study reported that the rate of arterial dissection was higher in female subjects (3.3%) in comparison to male subjects (2.4%) [29]. Procedures complicated by arterial dissection were most commonly indicated for CTLI and involved intervention of the femoropopliteal artery [30]. Specifically following intervention of the femoropopliteal artery, arterial dissection was reported to be more common among female patients (53.2% of patients) in comparison to male patients (44.8%). 

The need for reintervention following the original endovascular treatment because of complications was considered in several studies. Overall, female subjects had a higher likelihood of requiring reintervention (average being 15.75%), versus male patients (average being 12.55%) [31,35,36]. At one-year post-intervention, the studies revealed discrepancies whether males or female patients were at greater risk. Two studies suggested that male subjects had a higher risk of reintervention, while one study noted that female subjects had a higher risk for reintervention [31,35,36]. At two years post-intervention, female patients had a lower likelihood of reintervention-free survival (65%) in comparison to male patients (73%) [22]. At four years post-intervention, female patients also had a lower likelihood of reintervention-free survival (68.8%) in comparison to male patients (75.1%) [36]. 

As for the presentation in patients requiring reintervention, claudication was the most common presenting symptom needing intervention (35.4%) compared to CTLI (30.4%) [36]. Furthermore, the femoropopliteal region was the most common site requiring reintervention among female patients [32,33]. For female patients requiring reintervention, one study found that stent use was less likely to require reintervention than angioplasty [32]. For female patients who did undergo angioplasty, a different study found that plain old balloon angioplasty (POBA) was less likely to require reintervention (27.3%) in comparison to drug-coated balloon angioplasty (DCB) (34.6%) [36].

Variations in Findings 

Three studies reported findings of lower overall mortality and reduced rates of complications, including amputation, among female patients following endovascular treatments for PAD [29,33,35]. Other studies found that female patients had higher risks of periprocedural complications, including arterial dissection, bleeding complications, and access site hematomas [29-31,33]. Higher rates of complications were also found in female patients in multiple studies, including limb amputation, cardiac and pulmonary complications, and a need for reintervention one year after the original intervention [29-32,35].

Meanwhile, three studies, reported differing sex-specific risks of amputation following endovascular intervention. One study found no risk of amputation following intervention (2.4% risk for both male and female patients), whereas two other studies suggested that male patients had a higher risk of amputation following intervention (average being 8.95%) in comparison to female patients (average being 5.4%) [29,33,36]. For both male and female patients, the tibial artery was more likely to undergo major amputation in comparison to the femoropopliteal artery [36]. In addition, for both male and female patients, CTLI was more likely to be the indication for patients to undergo major amputation following endovascular intervention in comparison to claudication [36].

Furthermore, studies differed in highlighting the regions that were more likely to undergo intervention and the method used. Two studies found that female patients were more likely to undergo intervention of the femoropopliteal region, while three found the iliac region was more common [29,31,32,35,36]. Concurrently, two studies found that atherectomy was the most common intervention used in PAD in female patients, while three others found use of stents were more common [29,32,35-37].

Overall, female patients with PAD had increased rates of complications following endovascular treatment [29-32,36]. Although a few studies did report an increased prevalence of complications among male patients, they found that female patients had higher rates of certain complications, including reintervention [33,35]. Several studies that investigated the rates of amputation following endovascular treatment found that males and female patients had a similar prevalence [31,36].

Discussion

This scoping review identified important sex-based differences in patient characteristics, procedural indications, intervention patterns, and outcomes following endovascular treatment for PAD. Overall, female patients appeared to present with more advanced disease, experienced greater procedural complexity, and had higher rates of complications and reintervention, although findings were not fully consistent across studies. Female patients were more frequently treated for complete vessel occlusion and were more likely to present with CLTI, whereas male patients more often presented with claudication and stenotic disease. This pattern suggests delayed recognition and referral in female patients, potentially leading to more advanced disease at presentation. Contributing factors may include atypical symptom presentation, diagnostic overlap with musculoskeletal conditions, and under-recognition of PAD in women.

Baseline characteristics also differed by sex. Female patients undergoing intervention were more often older, of African American descent, and non-smokers, with higher rates of obesity, anemia, COPD, and functional impairment. In contrast, male patients carried a higher burden of traditional atherosclerotic risk factors, including smoking, diabetes, and coronary artery disease, suggesting that PAD risk profiles in female patients may be less fully captured by conventional cardiovascular risk models.

Procedural patterns varied across studies, with no clear consensus on the most frequently treated vascular territory or intervention type. Both femoropopliteal and iliac segments were commonly reported, and stenting and atherectomy were the most frequently described interventions, likely reflecting heterogeneity in populations, lesion characteristics, and operator preference. Female patients generally experienced higher rates of peri-procedural complications, including bleeding, hematoma, distal embolization, arterial dissection, and access site thrombosis, as well as higher reintervention rates and lower long-term reintervention-free survival. These findings may reflect both biological factors, such as smaller vessel caliber and diffuse disease, and procedural challenges. Findings related to amputation and mortality were inconsistent, with some studies showing no sex differences and others reporting higher risk in male or female patients. Generally, CLTI and tibial artery involvement were consistently associated with worse outcomes in both sexes.

Overall, while female patients with PAD appeared to present with a more advanced disease phenotype and experience a higher burden of procedural complications and reinterventions, the evidence remained inconsistent, with no clear or uniform sex-based disparities following endovascular intervention for symptomatic PAD. These discrepancies may reflect a combination of factors, including under-recognition of symptoms in female patients, differences in diagnostic and treatment pathways, and the limited volume of sex-specific research in this area.

Limitations

One of the greatest limitations of this scoping review was the heterogenous data regarding PAD intervention. This scoping review identified heterogeneous data regarding PAD intervention outcomes in the US, including variability in study design, patient populations, outcomes and follow up durations, which limits direct comparison of complication and reintervention rates across studies. This heterogeneity highlights significant gaps in the literature regarding sex-specific complications and risk. The creation of a larger database of primary data regarding outcomes of these procedures, would allow for greater analysis of potential risk factors such as sex. In addition, sociocultural influence on the PAD outcome should be further investigated. Given that female patients consistently present with diverse symptoms, there may be a need to find new ways to screen for PAD.

Each study provided insight into the influence of comorbidities such as diabetes, obesity, smoking, ethnicity, etc., regarding the choice of treatment modality. Four studies also provided insight on the utilization of the Trans-Atlantic Inter-Society Consensus (TASC) score, a categorization of the severity of arterial lesions, regarding the choice of treatment modality [26,28,29,33]. Meanwhile, each study lacked any evaluation as to how comorbidities and lesion of severity might influence the outcome of endovascular procedures. In addition, no study compared the outcomes across each of the different modalities and which complications occurred, despite some studies comparing reintervention and outcomes between select treatment modalities [28,32]. Additionally, there was no data on the prevalence of specific outcomes among female patients with PAD after they underwent a specific endovascular intervention. 

Further limitations of this scoping review included minimal availability of articles that met the inclusion criteria, and those that did, had significant limitations including small sample size and limited observation period. All included studies were retrospective cohort designs. Therefore, the reported relationships between comorbidities, severity, and outcomes reflect associations rather than causal effects and may be influenced by confounding or selection bias. Methodological quality and risk assessment were performed using the JBI critical appraisal tools, yet the included studies possessed some methodological limitations, therefore the findings should be interpreted cautiously and would be inappropriate for the review to be used as evidence for clinical guidelines, limitations, and/or recommendations. Given that each of these studies were confined to the US, it is important to consider the socio-cultural influence of comorbidities and thus procedural outcomes. Additionally, with the continued development of artificial intelligence, its use could potentially be beneficial in screening female patients more effectively and efficiently to help mitigate potential long-term complications.

## Conclusions

Overall, the available literature suggests female patients with PAD may have a higher risk of complications requiring re-intervention after endovascular intervention. However, interpretation of these findings is limited by the heterogeneity of the included studies and the absence of quantitative synthesis. Current literature regarding sex-based outcomes of endovascular treatment of PAD in the US remains limited. It is necessary to have studies that analyze the outcomes among male versus female patients to identify variables that may impact procedural complications and outcomes. Factors contributing to a potential discrepancy, which were not explored in this scoping review, include anatomical differences between male and female patients, higher basal levels of inflammation in female patients, physician bias, misinterpretation of symptoms of female patients, and other sex-based differences that may impact disease progression and/or the recovery process. Further research should be conducted to measure standardized outcome measures and sex-stratified analyses to raise more awareness for the sex-based outcomes of PAD.
